# Tube Feeding in Individuals with Advanced Dementia: A Review of Its Burdens and Perceived Benefits

**DOI:** 10.1155/2019/7272067

**Published:** 2019-12-19

**Authors:** Ezekiel Oluwasayo Ijaopo, Ruth Oluwasolape Ijaopo

**Affiliations:** ^1^East Kent Hospital University Foundation Trust, William Harvey Hospital, Ashford, Kent TN24 0LZ, UK; ^2^Royal Stoke University Hospital, Community Haywood Hospital, Stoke-on-Trent ST6 7AG, UK

## Abstract

**Background:**

Dementia remains a growing concern for societies globally, particularly as people now live longer. About 90% of individuals with advanced dementia suffer from eating problems that lead to general health decline and ultimately impacts upon the physical, psychological, and economic wellbeing of the individuals, caregivers, and the wider society.

**Objective:**

To evaluate the burdens and perceived benefits of tube feeding in individuals with advanced dementia.

**Design:**

Narrative review.

**Methods:**

Computerized databases, including PubMed, Embase, Medline, CINAHL, PsycInfo, and Google Scholar were searched from 2000 to 2019 to identify research papers, originally written in or translated into English language, which investigated oral versus tube feeding outcome in individuals with advanced dementia.

**Results:**

Over 400 articles were retrieved. After quality assessment and careful review of the identified articles, only those that met the inclusion criteria were included for review.

**Conclusion:**

Tube feeding neither stops dementia disease progression nor prevents imminent death. Each decision for feeding tube placement in individuals with advanced dementia should be made on a case-by-case basis and involve a multidisciplinary team comprising experienced physicians, nurses, family surrogates, and the relevant allied health professionals. Careful considerations of the benefit-harm ratio should be discussed and checked with surrogate families if they would be consistent with the wishes of the demented person. Further research is required to establish whether tube feeding of individuals with advanced dementia provides more burdens than benefits or vice-versa and evaluate the impacts on quality of life and survival.

## 1. Background

Dementia remains a public health priority and a growing concern for societies globally, particularly as the number of people suffering from dementia is increasing [1]. Currently, 50 million people in the world have dementia, and these people are estimated to triple to over 150 million by 2050 [2]. In every three second, someone in the world develops dementia, thus leading to 9.9 million new cases of dementia annually [3]. Alzheimer's disease, which is the commonest form of dementia, has become the most feared disease in the United States ahead of cancer. It kills a lot more people in the US than breast and prostate cancer combined and also currently accounts for the most common cause of death in England and Wales [2].

Advanced dementia can be described as a state of worsening mental and physical capabilities decline in persons with dementia, thereby resulting in dependency in (basic) daily personal care needs, such as dressing and eating, and cause severe limitations in verbal communication [4–6]. Studies show that nearly 90% of people with advanced dementia have eating problems [7] which increase the risk for weight loss, malnutrition, and general health decline [8–11]. Dementia causes physical, psychological, and economic impacts upon the individuals, caregivers, and the society at large [12]. It is estimated that almost 82 billion hours of informal care are provided annually for people with dementia worldwide [2], and the huge economic impact is currently evaluated worldwide to cost one trillion dollars [13].

The rates of feeding tube placement in advanced dementia individuals vary across different countries. For instance, in the United States, a cross-sectional study involving 186,835 nursing home residents with advanced cognitive impairment reports that 34% of residents with advanced dementia in the nursing home had feeding tubes [14].

Tube feeding is an alternative feeding method for people with unsafe swallowing and hence cannot attain sufficient oral intake to maintain their body energy requirements [15]. According to the American Society for Parenteral and Enteral Nutrition, tube feeding is defined as an “enteral nutrition provided through a tube, catheter, or stoma that delivers nutrients distal to the oral cavity” [16]. The commonly used feeding tubes are the nasogastric (NG) and gastrostomy tubes which include percutaneous endoscopic gastrostomy (PEG) tube (considered the gold standard) and radiologically inserted gastrostomy (RIG) tube [17].

Discussing feeding options with family surrogates is often complex and emotive, raising not only clinical issues but also ethical ones for the healthcare practitioners [18–20]. Similarly, the religious beliefs, cultural background, and ethnicity of families often play significant roles in the surrogates' discussion with healthcare providers [21]. Some family caregivers may have the impression of being indirectly contributing to their loved one's starving when they fail to support enteral feeding for their loved ones with dementia.

While several professional societies and experts have recommended careful hand feeding as the standard/ceiling of care for all individuals with advanced dementia who experience difficulty with oral intake [5, 22–24], other experts have argued in support of feeding tube placement [25–29]. Quite justifiably, the debate continues as there has not been any randomised controlled trial done to compare the benefits and burdens of tube feeding interventions with oral feeding in people with advanced dementia due to ethical concerns. The guidelines and recommendations from some professional societies which discourage feeding tube placement in severe dementia patients are based only upon the available evidence from existing observational studies and some experts' opinions [22, 30].

However, healthcare practitioners are faced with discussing this complex and challenging issue almost on a regular basis with the surrogate families and caregivers. Unfortunately, recent studies also show persistent knowledge gap among healthcare professionals regarding tube feeding of patients with advanced dementia [31]. The lack of appropriate evidence-based knowledge about tube feeding results in providing second-rated information to patient's families [32, 33]. This study explores the databases for studies conducted in the last two decades to review the current knowledge on tube feeding of individuals with advanced dementia and discusses its burdens and perceived benefits. It also provides opportunity for increasing knowledge and awareness for healthcare professionals and the public, particularly the family caregivers involved in caring for individuals with advanced dementia at homes or health institutions.

## 2. Methods

Computerized databases, including PubMed, Embase, Medline, CINAHL, PsycInfo, and Google Scholar were searched from 2000 to 2019 to identify scientific research papers, originally written in or translated into English language. Broad search (MeSH) terms used were “enteral feeding in dementia,” “tube feeding in dementia,” “artificial nutrition in dementia,” “burdens of tube feeding in dementia,” “benefits of enteral feeding in dementia,” and “percutaneous endoscopic gastrostomy tube feeding in dementia.” Over 400 articles were retrieved. Only studies that investigated the burdens and/or benefits of tube feeding versus oral feeding in individuals with advanced dementia were selected for review. Other studies on tube feeding that included dementia subjects among other medical conditions were also assessed, particularly if data for the dementia subjects were provided in the studies. Citations from all the relevant studies were also reviewed to obtain additional publications. Data synthesis and conclusions for this study came from available evidence obtained from the studies reviewed.

## 3. Burdens of Tube Feeding in People with Advanced Dementia

When an individual with advanced dementia experiences difficulty with eating or persistently refuses foods and their advanced wishes are unknown, surrogate families are often faced with making decision regarding what option of feeding route is best appropriate for maintaining nutritional status and survival. Should placement of a feeding tube or a careful hand feeding be considered as the ceiling of care? Mitchell et al. claimed feeding tube insertion rates among people with late-stage dementia in the US nursing home from 2000 to 2014 declined from nearly 12% to 6%, respectively [34]. While it is true there are declining trends in the placement of feeding tubes in advanced dementia, the practice still continues [35, 36].

The American Geriatrics Society [22] position statement insists that careful hand feeding is almost as good as tube feeding for the outcomes of comfort, aspiration pneumonia, functional status, and death while, at the same time, avoiding the burdens and complications associated with tube feeding. Some studies [37–39] also report that involving dieticians to prescribe dietary supplements in addition to the regular diets have proven to be effective in maintaining nutritional status in elderly people with advanced dementia.

A prospective study that compared clinical course and outcomes of 88 elderly demented patients with disabilities via their feeding mode found that tube feeding showed no beneficial effect on nutritional outcomes and failed to promote the healing of pre-existing pressure sores compared with oral feeding [40]. Similarly, a recent retrospective analysis of 392 patients claimed PEG insertion in patients with dementia failed to improve the nutritional status, rates of hospital readmission, or the short- and long-term survival when compared with PEG feeding among patients with other medical conditions such as stroke, motor neurone disease, and oropharyngeal cancers [41]. This latter study reinforces the outcome of an earlier systematic review of seven observational studies which declared there is insufficient evidence to conclude that tube feeding of individuals with advanced dementia is effective in improving survival, quality of life, or nutritional status and neither helps in promoting the healing of pressure sores [42].

The nonsuperiority of tube feeding over oral feeding is supported further in a recent study that evaluated the knowledge and perceptions of 168 physicians about PEG feeding in advanced dementia individuals. The authors report that 71% of the physicians believed careful hand feeding is almost as good as tube feeding for the outcome of comfort, and nearly half (49%) of them believed nutritional status rarely improves with tube feeding [43]. Comparatively, the general consensus among gastroenterologists in the USA is that PEG placement is not beneficial for patients with advanced dementia [44]. In fact, one study finds that nearly 20% of the tube-fed advanced dementia residents in the nursing home had their feeding tubes either replaced or repositioned during the 2 years of prospective follow-up, thereby resulting in more frequent visits to the emergency department. Worse still, nearly one-third (30%) of the demented residents who had their feeding tubes replaced needed at least two replacements, and the median survival time after a repositioning or replacement was 54 days [45].

### 3.1. Survival Burden

An 8 year (1999–2007) prospective study of 36,492 nursing home residents with advanced dementia and new eating problems that investigated whether individuals receiving PEG feeding had better survival compared with those without PEG found that PEG feeding, regardless of the timing of insertion, does not improve survival [46]. Another 18-month follow-up study that analyzed survival in older adults with dementia and eating problems, by comparing PEG-fed patients with those who were hand-fed, reported that survival was shorter in the PEG-fed group compared with the group that was fed orally. The authors further state that PEG feeding was associated with notable earlier mortality even after adjustment for likely confounders such as age, dementia type, and staging [47].

Besides PEG tubes, feeding people with late-stage dementia via an NG tube neither increases survival nor reduces aspiration pneumonia risk. Studies show that demented patients who receive NG feeding have much higher risk of death compared with those receiving oral feeding [48]. The mortality rate for tube-fed demented patients rose from 41.9% at 3 months to 58% at 6 months compared with those who had oral feeding, which rose from 11% to 28% at 3 and 6 months, respectively [48].

### 3.2. Aspiration Pneumonia Burden

The concern that a demented person who experiences repeated choking during meals has a high risk of developing aspiration pneumonia is among the most common indications for inserting feeding tube in the first place. Ironically, aspiration pneumonia remains a significant complication of tube feeding and frequently accounts for the cause of death after tube feeding [49, 50]. The occurrence of aspiration pneumonia, which may occur even when there is no clear evidence of vomiting, can be potentially life-threatening [51]. A prospective observational study that assessed aspiration pneumonia incidence in advanced dementia patients receiving enteral feeding reported that aspiration pneumonia occurred almost twice as frequently in individuals who received tube feeding compared with those who received oral feeding [48].

Also, many long-term care facilities have a common practice of stopping oral feeding/care of residents with advanced dementia soon after they are established on tube feeding [52]. As a result, tube-fed dementia patients have high tendency to suffer from the neglect of their oral health hygiene, thus leading to the colonisation of their oropharynx by pathogenic microorganisms which subsequently increase the risk of oral diseases and aspiration pneumonia [21, 53]. A study involving frail elderly patients who received tube feeding in nursing and skilled nursing facilities compared with those fed orally reports high prevalence of Gram-negative bacteria (*Pseudomonas aeruginosa*, *Klebsiella*, and *Proteus*) isolations in the tube-fed group compared with the patients' group that had oral feeding. The authors report 81% of patients fed via an NG tube, and 51% of patients fed via PEG as against 17.5% of patients who received oral feeding had Gram-negative bacteria cultured from their oropharynx [54]. The presence of these pathogenic Gram-negative bacteria in the oropharyngeal secretions obviously increases the risk for aspiration pneumonia [55].

### 3.3. Pressure Sores Burden

It is arguable whether tube feeding helps to prevent the development of new pressure sores or promotes the healing of the pre-existing pressure ulcers. A study by Arinzon et al. [56] reports that while tube feeding of very dependent and demented elderly in long-term care lead to improvements in blood count, renal function, electrolytes, and hydration status, it does not provide any benefit in preventing pressure sore development in the patients [56]. Another cohort study conducted using Minimum Data Set (MDS), obtained from national storage of demographic and clinical information of nursing home residents living in US-certified Medicare or Medicaid facilities, compared the records of residents with advanced cognitive impairments receiving tube feeding with those without PEG feeding. The study analysis showed that residents with PEG feeding were more than twice likely to develop a new pressure ulcer. In the similar manner, established pressure ulcers were less likely to heal or show improvement in advanced cognitively impaired residents who had PEG feeding compared with those without PEG feeding [46]. However, it is important to emphasize that the latter report should be used with caution as several experts have issued clear warning that using administrative data alone for research evidence do not provide the true reflection of outcome, particularly as the reported figures often have bias of overdocumentation [57].

### 3.4. Refeeding Syndrome Burden

Refeeding syndrome is another potentially life-threatening complication that may occur in individuals receiving artificial nutritional support via feeding tubes or parenteral nutrition, after a period of starvation [58, 59]. The burdens arising from refeeding syndrome are characterised by severe electrolyte imbalance and fluid retention that may cause various organs and systems failure, thus contributing to worsening morbidity and high risk of death [25, 58, 60].

### 3.5. Costs Burden

One observational study found the daily costs of hand-feeding individuals with advanced dementia in nursing home were higher compared with residents receiving tube feeding ($4219 vs. $2379). But, the total costs billed to Medicare were greater for the tube-fed residents ($6994 vs. $959) due to the high costs associated with the placement of feeding tubes and hospital admissions with or without the management of likely complications in the emergency department [61]. The Medicare costs for inpatient care among nursing home residents with late-stage dementia showed that one-year hospital costs were $2224 more expensive in nursing home residents with feeding tubes than those without tube feeding [62]. More so, nursing home residents with advanced dementia receiving tube feeding have higher odds of spending more time in intensive care units and tend to acquire more healthcare costs for treating associated complications related to feeding tube placement [62]. Whether or not the cost burden issue provides a valid point to discourage feeding tube placement in advanced dementia patients is another controversial topic, particularly as healthcare systems operate differently across the regions of the world. For instance, in countries where the healthcare finance budgets are limited or where individuals or third-party have to pay medical bills, healthcare cost-benefit analysis may play significant role in decision-making, especially where feeding tube insertion is deemed questionable to benefit the individual with severe dementia.

### 3.6. Other Burdens and Complications

It is not unusual to see family members feeling unease and express concerns that their demented loved ones are too frail or too old to undergo surgical procedure/operation for feeding tube placement [63]. They may see it as causing unnecessary suffering and burden for the demented individuals. Also, there is serious worry of possible needs to use physical restraints and sedative drugs to prevent patient from pulling out feeding tube due to dementia-related agitation and lack of cognition [50, 64]. Studies have shown that nearly two-thirds of nursing home residents get agitated and pull their feeding tubes within the first two weeks of insertion [65]. Also, there is disconcertment that PEG-fed demented individuals are going to be deprived the pleasure of eating as well as the natural human interactions that come with oral feeding [64].

Other burdens that are directly related to the PEG placement may include concerns about peristomal wound leakage, infection risk, tube leaks or blockage, local pain and bleeding, colonic fistulae, as well as sepsis from abdominal abscess and potential death [51, 66, 67]. Several formulations of the enteral feeds may cause gastrointestinal symptoms such as abdominal discomfort, diarrhoea, and constipation which can contribute to the discomfort experienced by the demented individuals [49–51]. The diarrhoea problem is a major concern and accounts for the most common gastrointestinal side effect in patients receiving tube feeding. Its causes are multifactorial and may occur in a wide range (2% to 95%) of patients receiving tube feeding [68, 69]. Also, fluid overload complication resulting in pulmonary oedema and swelling of extremities is another trouble that can occur from tube feeding [50]. A survey study of home healthcare nurses in United States that explored their perceptions regarding suffering, artificial nutrition, and hydration in advanced dementia reports that artificial nutrition and hydration prolonged patients' suffering due to burden of the procedure, the need for restraints, and increase chances of developing fluid overload complications [70]. Other studies also report significant higher risk of in-hospital mortality after PEG placement [71–73]. A 30-day mortality risk in tube-fed individuals with advanced dementia may vary from as high as 20% to 40% [43, 74].

It is established that the majority (nearly 75%) of nursing home dementia residents receiving tube feeding had their feeding tube inserted during an acute hospital admission [45, 75]. Among the factors that often pressured physicians to agreeing to feeding tube placement include lack of awareness of appropriate evidence-based knowledge on tube feeding; cultural values and clinical practice that encourage family-oriented end-of-life decision-making; fear of litigation from possibly disallowing treatment that is potentially life-sustaining; emotional uneasiness to allow death by “starvation;” and the remuneration factors associated with the choice of PEG [50, 76]. More so, a recent evidence from multicentre study involving three tertiary and four community hospitals in New York that explore the opinion of physicians on PEG feeding in late-stage dementia shows that 63% of physicians claimed that families and surrogate decision-makers insisted on PEG placement even when the physician would not approve of it [43].

Besides the dementia disease progression, correctable causes of poor oral intake in patients with advanced dementia may include the following: oral health-related problems [13]; over sedation or loss of appetite from polypharmacy or side effects of drugs [77, 78]; infections (such as respiratory or urinary tract); comorbid medical conditions; constipation; depression or anxiety; and distress from other neuropsychiatric symptoms of dementia [5]. In addition, environmental stressors such as lighting, loud noise, extreme temperature, colours, and crowding are among other things that could cause irritations and discomfort, thus altering oral intake in severe dementia patients [79]. Therefore, older adults with advanced dementia presenting with eating problems should undergo proper evaluation to rule out these treatable conditions as the cause of the poor oral intake [5].

### 3.7. Burdens Relating to End of Life

It is important to emphasize at this point that individuals with advanced dementia may decline to eat or drink as they approach end-of-life period. Refusing food and fluid is a normal part of the natural dying process, particularly as the body slowly shuts down. Simple strategies that promote comfort by relieving dry mouth through oral care delivered by well-trained staff, as well as hand feeding as tolerated should be the main focus of care in these individuals [13, 22, 23, 64, 80]. Family surrogates and caregivers should be given adequate counselling and properly educated about what constitutes essential care needs for dying persons so as to help mitigate their distress and uneasiness at that difficult time.  Table 1 provides the summary of studies on the burdens of tube feeding in individuals with advanced dementia.

## 4. Perceived Benefits of Tube Feeding in Advanced Dementia Individuals

In the last two decades, many published studies have expressed different views on whether or not the insertion of feeding tubes in individuals with advanced dementia provides more harms than benefits or vice-versa [25, 27, 40, 81–89]. Obviously, this issue continues to generate widespread debate among experts in the care of the elderly medicine [25, 26, 28].

While it is strongly recommended that no individuals including people with advanced dementia should be force-fed, tube feeding provides a safer way of administering foods and fluids to maintain the nutritional status of people categorised as having unsafe swallowing [14, 90, 91]. NG tube is rarely considered an option in people with late-stage dementia due to lack of cognition and intolerance [5, 92]. Evidence also shows that PEG feeding is associated with a lower incidence of aspiration when compared with NG tube feeding [93]. Where NG tube feeding is in place, it is often recommended that its use for feeding should not go beyond 4 weeks due to associated higher risks of tube dislodgement, aspiration pneumonia, and difficulty with diet infusion [94].

Several factors that are independently associated with feeding tube placement in advanced dementia are known to include: younger age, male gender, ethnic minorities, lack of advanced directives, and no DNR (do not resuscitate) order [17]. Likewise, the type of dementia and associated comorbidities play significant roles as well. For example, nursing home residents with Alzheimer's dementia and vascular dementia with a background history of stroke are more likely to have tube feeding, whereas individuals with comorbidity of cancer are less likely to have tube feeding [17].

Dementia illness is overwhelming not just for the affected individuals, but also their family and caregivers. Quite often, family caregivers experience tension and anxiety around mealtimes [95]. Unfortunately, the mealtime period become unnecessarily prolonged and distressing for the family caregivers as great deal of time, effort, technique, and patience spent in providing assistance with hand feeding may produce almost zero success [25, 96]. Consequently, these family caregivers become worried that their loved ones may be starving to death from poor oral intake due to difficulty in coping with the challenges of food refusal by their demented relatives. It is therefore, not uncommon for these caregivers to have high expectations of benefit from feeding tube placement and view its intervention as a representation of high-quality care to address the feeding problems [50]. One study that assessed the surrogates' expectations of benefit from feeding tube placement found that 79% of surrogates believed tube feeding would improve patients' comfort, and 87% of them anticipated better quality of life. More than half (56%) of surrogates also felt tube feeding would provide better independence for patients [97]. Similarly, a recent study that investigated the opinions of physicians and nurses about artificial nutrition in individuals with advanced dementia report that nearly 80% of physicians supported the administration of artificial nutrition when life expectancy is between one month and six months, and about 70% of the nurses also supported the idea [98].

Even though there is general consensus that careful hand feeding should be offered to all individuals with advanced dementia who experience eating problems, significant concern arises when the demented person persistently refuses all forms of assisted hand feeding. While eating problems may indicate that advanced dementia has worsened and that the individual has entered the final phase of the dementia illness, clinically, this may not necessarily mean that end of life is imminent or that feeding tube placement will be futile or harmful to the individual. A systematic review of 9 studies conducted in 2015 which evaluated the outcomes of enteral nutrition for people with advanced dementia claimed no harmful outcomes were reported with tube feeding of individuals with advanced dementia when compared with persons without dementia [27].

### 4.1. Aspiration Pneumonia Incidence

A study of elderly Japanese patients with dementia who were fed via PEG showed evidence of reduced incidence of aspiration pneumonia with prolonged survival rate of more than two years compared with dementia patients fed via the nasogastric (NG) tube [99]. Another retrospective analysis that evaluated 58 severe dementia patients across nine psychiatric hospitals found that tube feeding helps to decrease the frequency of aspiration pneumonia and use of intravenous antibiotics and also prolongs median survival times by 23 months compared with dementia patients without tube feeding who had median survival times of two months [100]. The benefit of tube feeding in reducing aspiration pneumonia occurrence is further reinforced by recent survey of doctors' knowledge and attitudes about tube feeding in late-stage dementia. The survey reveals that 61.7% of the doctors claimed tube feeding prevents aspiration and more than half (51.7%) of the participants believed tube feeding prevents pneumonia [101]. The latter findings bolster the result of an earlier survey involving 195 primary care physicians in the United States that claimed PEG feeding of individuals with advanced dementia provides a range of benefits; 76% of the physicians agreed that PEG feeding reduce aspiration pneumonia and 61% of them believed it prolongs survival [74].

Even though clinical evidence shows tube feeding does not prevent the occurrence of aspiration pneumonia, however, when feeding tube is properly inserted, along with good tube care, and the feeding method is dexterously performed, the risk of vomiting, regurgitation, and aspiration is markedly reduced [53].

### 4.2. Nutrition and Survival Benefits

Quite commonly, older adults with advanced dementia suffer from recurrent hospital admissions which cause great concerns for the families. The recurrent hospitalisations mostly occur from poor oral intake which results in dehydration and subsequently leading to acute kidney injury (AKI) or acute-on-chronic renal impairments [102–104]. More so, patients may present with lethargy, worsening agitation and confusion, and systemic deterioration as a result of the electrolyte imbalance from dehydration [105].

However, one retrospective study that reviews the effectiveness of PEG feeding for nutritional support in patients with dementia finds that PEG feeding improves low serum albumin and other serum markers of malnutrition, hence preventing dehydration, and ultimately resulting in better clinical outcome [90]. It is therefore not surprising that the earlier study by Shega et al. [74] claimed 93.7% of primary physicians believe PEG feeding improves nutritional status in advanced dementia. Another prospective-based study that evaluated the global impact of PEG feeding on 60 elderly patients including patients with advanced dementia found significant reduction in the emergency department visits and hospital admissions, particularly in the following 6 months after PEG feeding was started compared with the preceding six months before PEG feeding [106]. The authors further report that PEG feeding improved biochemical markers (haemoglobin, albumin, and total proteins) which reflected better nutrition and hydration in the patients [106].

While it is true that tube feeding does not provide cure for the underlying swallowing difficulty in late-stage dementia, family caregivers and healthcare providers (that support tube feeding idea) maintain that PEG feeding helps to mitigate weight loss, sustain nutrition, and reduce the suffering that occurs from dehydration or malnutrition [28, 74, 107].

Regarding survival, a study by Shintani compared survival periods of advanced cognitively impaired elderly who received oral feeding versus PEG feeding. The author claimed the survival periods in those who received PEG feeding (736 ± 765 days) were nearly as twice as the elderly who had hand feeding (399 ± 257 days) [108]. Comparably, another recent study by Takayama et al. [109] claims that dementia patients who received tube feeding had longer median survival times of 695 days compared with those without tube feeding who had median survival times of 75 days [109]. Takayama et al. also reported that about 75% of the dementia patients with tube feeding survived more than a year, and nearly 50% of them survived more than two years [109].

While identifying factors that influence survival in older adults with advanced dementia following PEG tube insertion, a cohort study reports advanced age and higher baseline serum albumin as strong predictors of mortality and survival, respectively [110]. Patients with higher serum albumin level at baseline and a stable/increased serum albumin level during follow-up had better survival and improved quality of life one year after PEG tube insertion compared with patients who had lower serum albumin levels [110]. Correspondingly, a rise in serum albumin level ≥3.0 g/dL within six months after PEG placement has been found to contribute to survival and essential to having long-term survival [111, 112].

In addition to advanced age and low serum albumin levels, other determinants of poor prognosis in older adults with late-stage dementia before or after PEG tube placement have been reported to include physical dependence and significant comorbidities such as heart failure, chronic pulmonary airway disease, diabetes, and malnutrition [52, 89, 113–116].

### 4.3. Comparison of Tube Feeding in Advanced Dementia with Other Diseases

Individuals who are suffering from advanced/progressive neurological disorders (such as lateral amyotrophic sclerosis, Parkinson's disease, and stroke) and terminal illnesses including cancer, end-stage heart failure, and renal disease have shown demonstrable benefits from PEG placement for nutritional supports [49, 90, 117–121]. However, since tube feeding is not an absolute contraindication in individuals with advanced dementia, the question then arises as to why PEG feeding should be discouraged in this population group, particularly if there are no significant differences in the burdens of PEG feeding in demented people compared with people who have other medical conditions. A study that compared survival outcomes of 311 elderly patients with and without dementia after PEG placement reports that 12-month survival or mortality was not significantly different in patients with dementia and those without dementia [113]. More so, a recently published retrospective review that evaluated burdens and complications associated with tube feeding claimed no difference was found between the incidence rates of mechanical, gastrointestinal, or metabolic complications in patients with advanced dementia compared with those without dementia [122]. The authors further report that there is no evidence to support that tube feeding led to poorer prognosis or low survival in patients with dementia compared with those without dementia [122].

In fact, a survival comparison study by Malmgren et al. after PEG insertion in 191 older adults with different medical conditions found patients with dementia or Parkinson's disease have longest median survival times (244 and 233 days, respectively) compared with patients that had amyotrophic lateral sclerosis and malignancy of head and neck who had shortest median survival times of 75 and 106 days, respectively [123]. One recently published (Pih et al.) study also reports that patients with neurologic disease including dementia have much lower incidence of 30-day mortality post-PEG compared with patients with stroke and malignancy [124]. It is important to mention that this review could not establish whether the included dementia patients in the studies of Malmgren et al. and Pih et al. had moderately severe or advanced dementia illness as the patients' dementia stages were not specified.

### 4.4. Other Reported Benefits

In addition to maintaining the nutritional needs of individuals with advanced dementia experiencing eating problems, feeding tube provides a reliable route for administration of essential medications [52, 94]. Studies also reveal that majority of family members and caregivers of PEG-fed individuals with advanced dementia express psychological relief [70] and report being satisfied with the quality of life of patients [52]. Quite considerably also, the associated mortality relating to direct PEG procedure is low (1-2%), and complications are trivial [90].  Table 2 provides the summary of studies on the benefits of tube feeding in individuals with advanced dementia.

## 5. Implications for Practice

Generally, when decision is to be made about feeding tube placement in an individual with advanced dementia, careful care should be taken to avoid applying the same blanket rule guidelines and recommendations from professional societies which discourage feeding tube insertion to everyone with advanced dementia. The lack of randomised controlled trials on this topic due to ethical reasons makes the existing scientific evidence to be inconclusive. Hence, the current guidelines based mostly upon experts' recommendations and the existing observational studies whose study design and overall quality have been questioned need to be interpreted with caution [28, 30]. These guidelines have been criticised as overestimating the futility of tube feeding and understating its benefits [25–29].

While it may be clinically evident in certain individuals with advanced dementia that inserting the feeding tube will be burdensome and futile due to poor clinical conditions and frailty, it may, however, be beneficial for comfort and nutritional maintenance in other appropriately selected individuals who may not be clinically compromised, in order to help achieve the care plan goals [28]. The decision for PEG placement in late-stage dementia should be made on a case-by-case basis, after considering the evidence supporting potential benefits versus the substantial burden that tube feeding may constitute for the individual. Each decision-making process should involve multidisciplinary team meeting comprising experienced physicians, nurses, dieticians, speech and language team, as well as family members.

## 6. Conclusion

It is imperative that all healthcare professionals caring for individuals with advanced dementia always check or review advance directives with family surrogates and implement patients' wishes in the care plans [125]. However, in the absence of advance directive or any known preference of a demented patient, physicians should take active role in addressing the concerns of family surrogates and aim to provide appropriate information that will aid decision-making about feeding options. Regrettably, many surrogate families claim they seldom have their informational needs completely met by the healthcare providers [126]. Research evidence shows that when healthcare professionals use a structured decision aid [127] to provide evidence-based information to families or surrogates about feeding options, there is proven evidence of significant improvement in the quality of decision-making by the families or the surrogates [24]. The optimum goal of healthcare practitioners should be to assist the families and caregivers in making informed decision which is in the best interest of the demented individual, particularly as it relates to the person's comfort and quality of life.

Very importantly, since evidence shows that majority of the decision-making for feeding tube placement occur during acute care hospitalization, hospital doctors in acute care settings (including allied health professionals and primary care physicians) should be targeted for interventions that will help update their knowledge on the appropriate evidence-based practice relating to the use of tube feeding in individuals with advanced dementia. This will help to keep them well-informed and properly positioned to educate families and caregivers about the risks and potential benefits of tube feeding and ultimately provide better end-of-life care for patients with advanced dementia where necessary [128]. In addition, the medical staffs in acute care settings should be educated and informed to routinely seek the consult of geriatrics professionals for second opinion since the latter have much more experience in dealing with dementia patients.

It should also be emphasized that the most available research evidence and experts' recommendations agree that careful hand feeding is the recommended standard of care for older adults with advanced dementia [5, 22, 47]. However, where family caregivers have reluctance or feel differently about careful hand feeding, a thorough evaluation of the risks and benefits of tube feeding should be expounded. The benefit-harm ratio of feeding tube intervention should be discussed and checked with surrogate families if they would be consistent with the demented person's wishes. While these burdens and their perceived benefits are elaborated in this review, the overall impact of tube feeding on the quality of life of severely demented elderly remains unclear. This review agrees with the study of Jaul et al. [40] that following thorough discussion with surrogate families on the risks and benefits, tube feeding should not be antagonised when it is in line with the family's wishes and values. However, family caregivers should be clearly informed that tube feeding cannot stop dementia disease progression nor prevent imminent death.

Ultimately, a trusting relationship should ensue between the healthcare team and the family caregivers such that person-centered care plans detailing the individual needs and goals are clearly agreed upon and documented. Also, if a general consensus is reached such that insertion of feeding tube may be beneficial for a patient with advanced dementia, regular periodic reassessments should be done for prompt recognition and immediate management of complications that are directly related to tube feeding to help optimize comfort and reduce overall morbidity and mortality. Notwithstanding, the patient's care plan goals should include documentation for potential removal of feeding tube once the clinical evidence shows the burdens and complications of tube feeding outweigh its benefits. It is therefore, important to involve the palliative care team specialists for advice with comfort care for patients and provide needed supports for the family.

Further research, particularly ethically modified randomised controlled trials, is required to establish whether tube feeding of individuals with advanced dementia provides more burdens than benefits or vice-versa, and evaluate the impacts on quality of life and survival.

## Figures and Tables

**Table 1:** Summary of studies on the burdens of tube feeding in individuals with advanced dementia.

Article	Participants	Study design (follow-up)	Aim/objective	Outcome/conclusion
Jaul et al. [40]	88 patients (26 fed orally; 62 fed via NG tube)	Prospective survey study (17 months)	Compared the clinical course and outcome of elderly demented patients with severe disabilities via feeding mode	Tube feeding showed no beneficial effects on nutritional outcome in elderly patients with advanced dementia and does not aid the healing of pre-existing pressure sores as compared with oral feeding. The mean number of pressure ulcers in the tube and orally fed groups at the start to end of study were 1.05 to 0.97 vs. 2.28 to 1.92 (*P*=0.05 to 0.03), respectively

Ayman et al. [41]	392 patients	Retrospective analysis (48 months)	Compared rehospitalisation and mortality rates after PEG placement in dementia patients (165) versus stroke patients (124) and other patients' group with head and neck cancers and motor neuron disease (103)	PEG insertion did not reduce rehospitalisation rate at 6 months postprocedure in dementia patients compared with patients who had PEG for other condition (OR: 2.45 in the dementia group, 1.86 in the stroke group, and 1.65 in patients with oropharyngeal cancers and motor neuron disease; *P* < 0.05); also, mortality was higher in the dementia group (75%) within the first year after PEG placement compared with the stroke group (58%) and group C (38%) (*P* < 0.001)

Gieniusz et al. [43]	168 internal medicine physicians	Multicentre mixed-mode survey (none)	Evaluated physicians' knowledge and perceptions regarding PEG placement in individuals with advanced dementia	81% and 85% of physicians believed PEG placement does not increase survival nor reduce aspiration pneumonia, respectively; 71% and 61% of physicians claimed careful hand-feeding of advanced dementia people are nearly as good as tube feeding for the outcomes of comfort and functional status, respectively

Kuo et al. [45]	97,111 nursing home (NH) residents (5,209 had PEG; 91,902 had no PEG)	Secondary analysis of minimum data set (MDS) (2 years)	Assessed the natural history of feeding tube insertion in NH residents who followed-up for 2 years to measure their health care use and survival	Feeding tubes placement was associated with poor survival. 19.3% of residents who had feeding tube placement needed tube replacement or repositioning within 145 days after insertion, and the median survival was 54 days after replacement; also, one-year mortality after feeding tube insertion was 64.1% with a median survival of 56 days after insertion
Teno et al. [46]	4421 patients (1585 PEG-fed and 2836 Non-PEG-fed)	Propensity-matched cohort study (1 year).	Assessed benefits and risks of PEG feeding in the prevention and healing of a pressure ulcer in NH residents with advanced cognitive impairment (ACI)	NH residents who received PEG feeding were 2.27 times at higher risks of developing new pressure sores (95% CI 1.95–2.65) and had less odds of having their established pressure ulcer heal (OR 0.70, 95% CI 0.55–0.89)
Ticinesi et al. [47]	184 patients	Prospective observational study (18 months)	Compared survival rates and hospital readmissions in elderly demented patients who were PEG-fed versus those orally fed	At the follow-up, after adjustment for possible cofounders, mortality was higher in PEG-fed patients than orally fed patients, 70% vs. 40%, respectively (*P*=0.0002); however, hospital readmission rates during follow-up were insignificantly different in both groups (40% (PEG-fed) vs. 38% (orally fed), age- and sex- adjusted *P*=0.88)
Cintra et al. [48]	67 patients	Prospective nonrandomised observational study (6 months).	Compared hospital admissions, survival rates, and aspiration pneumonia incidence in dysphagic dementia patients on oral feeding versus alternative (mostly NG tubes) feeding route	No significant difference in number of hospital admissions in both groups (*p*=0.365); however, the incidence of aspiration pneumonia is twice as high in the alternative feeding group (RR: 2.32; 95% CI 1.22–4.40) Mortality at 3 months was 11.1% among the oral feeding group compared with 41.9% among the alternative feeding group (RR: 3.77; 95% CI 1.35–10.39) At 6 months, mortality was 27.8% in the oral feeding group versus 58.1% among the alternative feeding group (RR: 2.09; 95% CI 1.14–3.83)
Leibovitz et al. [54]	215 patients	Cross-sectional comparative study (not clear)	Compared the pathogenic oral floral colonisation risk in tube-fed elderly patients (*n* = 135) with their orally fed counterparts (*n* = 80) in skilled nursing facilities	Tube feeding is correlated with pathogenic organisms' colonisation of the oropharynx; Gram-negative bacteria were isolated in 81% of patients fed via the NG tube and from 51% of the PEG-fed patients as against 17.5% in the orally-fed patients (*P* < 0.0001); no correlation was found between the duration of tube feeding and bacterial isolations
Arinzon et al. [56]	261 patients	Prospective study (21 months before and after analysis)	Evaluated the effectiveness of enteral nutrition in improving survival, nutritional, and functional status of the very dependent demented elderly patients	Although the tube-fed group had improvements in blood count, renal function, and electrolyte and hydration status, the mortality rate was higher in the tube-fed group (42%) than in the control group (27%, *P* > 0.05). Also, nutrition-related complications were higher in the tube-fed group than in the orally fed (control) group, 61% and 34%, respectively

**Table 2:** Summary of studies on the benefits of tube feeding in individuals with advanced dementia.

Article	Participants	Study design (follow-up)	Aim	Outcome/conclusion
Nunes et al. [90]	46 patients	Retrospective study (80 months)	Examined the effectiveness of PEG feeding for nutritional support in patients with dementia	PEG feeding improves low albumin including the serum markers of malnutrition and poor clinical outcome; serum albumin levels (95% CI: 3.3–3.6; *P* < 0.01) and transferrin levels (95% CI: 182–206; *P* < 0.05) were significantly improved after 3 months of PEG feeding; high albumin, transferrin, and cholesterol levels at admission were positively correlated with survival Mean and median survivals after PEG placement were 21 and 18 months, respectively

Giantin et al. [99]	261 patients (155 PEG-fed; 106 NG tube-fed)	Survey study (6 months before and after analysis)	Clinical evaluation of elderly Japanese patients with dementia who underwent PEG feeding versus NG- tube feeding	Survival rates among PEG-fed patients were 27 months higher than those fed via NG tubes (mean (SD): PEG group, 65.6 (5.6%) versus NG tube group, 44.4 (9.8%); *P*=0.019) PEG feeding provided evidence of reduced incidence of aspiration pneumonia when compared with NG tube feeding

Takenoshita et al. [100]	58 patients (46 with tube feeding and 12 without)	Retrospective study (60 months)	Evaluated the frequency of pneumonia before and after tube feeding in severe dementia patients	Tube feeding decreased pneumonia and antibiotic use in patients with severe dementia compared with those without tube feeding Tube feeding was associated with significantly longer survival (hazard ratio 9.8, 95% CI 3.6–27.0, *p* < 0.001); advanced dementia patients on tube feeding had median survival times of 23 months compared with median survival times of two months among those without tube feeding
Cúrdia et al. [106]	60 patients (26 dementia; 18 stroke; 5 head injury; 3 anoxic encephalopathy; 2 ALS; 3 other conditions)	Prospective study (24 months)	Analyzed the global impact of PEG feeding in patients followed-up in specialised multidisciplinary PEG clinic	6-month period after PEG placement showed significant decrease in the mean number of emergency department visits compared with 6 months before PEG insertion (1.1 vs. 2.2; *P*=0.003) as well as the mean number for hospital admissions (0.3 vs 1.4; *P* < 0.001). respectively 53.8% of patients with pre-existing pressure ulcers had complete healing after PEG placement at 6-month follow-up PEG feeding improved biochemical markers (such as haemoglobin, albumin, and total proteins) that reflected better nutrition and hydration in the patients
Shintani [108]	80 patients	Retrospective study (5 years)	Compared survival periods of elderly patients with neurologic impairments in those receiving oral intake, PEG feeding or home parenteral nutrition	Survival periods of the advanced cognitively-impaired elderly receiving PEG feeding (736 ± 765 days) were nearly as twice that of the elderly adults having oral intake (399 ± 257 days); home parenteral nutrition survival was 736 ± 765 days
Takayama et al. [109]	185 patients (129 dementia; 44 schizophrenia; 6 mood disorders; 6 others)	Retrospective study (>1000 days)	Compared the survival times with or without tube feeding in patients with dementia or psychiatry disease	Median survival times were longer for dementia patients with tube feeding (695 days) compared With those without tube feeding (75 days *P* < 0.001) About 75% of the dementia patients with tube feeding survived more than a year, and about 50% of them survived more than two years
Higaki et al. [113]	311 patients	Retrospective cohort study (3 years)	Compared survival outcomes of elderly patients with and without dementia after PEG placement	Survival or mortality was not significantly different in the patients with dementia and those without dementia (*P*=0.62)
Orlandoni et al. [122]	585 patients	Retrospective observational study (5 years)	Compared the outcomes and harmful effects of home tube feeding in patients with advanced dementia and patients without dementia	No difference was found between the incidence rates of mechanical, gastrointestinal, or metabolic complications in patients with advanced dementia compared with patients without dementia No evidence to support that tube feeding led to poorer prognosis or low survival in patients with dementia compared with patients without dementia (*p* > 0.05).
Malmgren et al. [123]	191 patients (16 dementia; 95 stroke; 11 Parkinson's disease; 35 malignancy; 13 neurological diseases; 19 miscellaneous)	Retrospective study (5 years)	Evaluated the indications and survival after PEG insertion in patients older than 65 years	Overall median survival was 123 days, and 30-day mortality was 22% Patients with dementia or Parkinson's disease had the longest median survival, which was 244 and 233 days, respectively, while patients with other neurological diseases and malignancy had the shortest median survival, 75 and 106 days, respectively
